# Mechanisms for similarity matching in disparity measurement

**DOI:** 10.3389/fpsyg.2013.01014

**Published:** 2014-01-08

**Authors:** Ross Goutcher, Paul B. Hibbard

**Affiliations:** ^1^Psychology, School of Natural Sciences, University of StirlingStirling, Scotland, UK; ^2^Department of Psychology, University of EssexColchester, UK

**Keywords:** binocular vision, disparity measurement, correspondence problem, similarity, cross-correlation, binocular energy model

## Abstract

Early neural mechanisms for the measurement of binocular disparity appear to operate in a manner consistent with cross-correlation-like processes. Consequently, cross-correlation, or cross-correlation-like procedures have been used in a range of models of disparity measurement. Using such procedures as the basis for disparity measurement creates a preference for correspondence solutions that maximize the similarity between local left and right eye image regions. Here, we examine how observers’ perception of depth in an ambiguous stereogram is affected by manipulations of luminance and orientation-based image similarity. Results show a strong effect of coarse-scale luminance similarity manipulations, but a relatively weak effect of finer-scale manipulations of orientation similarity. This is in contrast to the measurements of depth obtained from a standard cross-correlation model. This model shows strong effects of orientation similarity manipulations and weaker effects of luminance similarity. In order to account for these discrepancies, the standard cross-correlation approach may be modified to include an initial spatial frequency filtering stage. The performance of this adjusted model most closely matches human psychophysical data when spatial frequency filtering favors coarser scales. This is consistent with the operation of disparity measurement processes where spatial frequency and disparity tuning are correlated, or where disparity measurement operates in a coarse-to-fine manner.

## INTRODUCTION

The retrieval of depth information from binocular disparity depends crucially on the correct linkage of corresponding image points between left and right eyes. Finding a successful, biologically plausible, solution to this correspondence problem has been a central task for researchers in binocular vision for many years (e.g., Marr and Poggio, 1979; Pollard et al., 1985; Jones and Malik, 1992; Qian and Zhu, 1997; Chen and Qian, 2004; Read and Cumming, 2007). While a number of constraints on possible matches have been proposed, based on the likely distributions of disparities in natural scenes (e.g., Marr and Poggio, 1979; Pollard et al., 1985; Zhang et al., 2001; Hibbard and Bouzit, 2005; Goutcher and Hibbard, 2010), one of the most important aspects in binocular matching is local image similarity.

Similarity-based matching is essential for any model that seeks to mimic the performance of human observers. Correspondence matching biases have been found based on similarity of contrast (Anderson and Nakayama, 1994; Smallman and McKee, 1995; Goutcher and Mamassian, 2005), contrast polarity (Watanabe, 2009), luminance (Goutcher and Hibbard, 2010), color (den Ouden et al., 2005), orientation, motion direction, and speed (van Ee and Anderson, 2001). Similar matching constraints have also been demonstrated in motion perception, for which an analogous matching problem exists (Hibbard et al., 2000). Such results provide compelling evidence to support computational assertions of the importance of feature similarity. The importance of similarity must, however, be matched against the need for flexibility in disparity measurement. Mechanisms for disparity measurement must be able to tolerate dissimilarity between matching features, which occurs frequently in natural images.

Dissimilarity of matching features is most obviously seen in the case of orientation. Differences in the orientations of matching proximal features will arise if the distal feature is slanted away from the horopter (i.e., slanted in depth, or present in the peripheral visual field). Models must, therefore, be able to tolerate some degree of orientation difference so as to encode these orientation disparities. Similarly, differences in luminance and contrast must also be tolerated. While luminance and contrast differences between eyes do degrade stereoacuity (Simons, 1984; Halpern and Blake, 1988; Schor and Heckmann, 1989; Reynaud et al., 2013), stereopsis is still viable. Indeed, such differences also appear to support distinct stereoscopic perceptions. When presented with vertically oriented squarewave grating patterns containing differences in luminance or contrast between the two eyes, observers report that individual bars are rotated in the depth plane, the so-called venetian blind effect (Cibis and Haber, 1951; Fiorentini and Maffei, 1971; Filley et al., 2011; Dobias and Stine, 2012).

Early models of correspondence matching sought to explicitly encode the properties of “image primitives” to allow for similarity-based matching (e.g., Marr and Poggio, 1979; Pollard et al., 1985). More recently, however, similarity-based matching has been implicit in models that measure disparity using cross-correlation (Banks et al., 2004; Filippini and Banks, 2009; Allenmark and Read, 2010, 2011) or cross-correlation-like (Fleet et al., 1996; Qian and Zhu, 1997; Chen and Qian, 2004) procedures. Such models encode similarity by cross-correlating spatially extended image patches. Using spatially extended patches means that local image regions with similar structures will elicit stronger responses. Note, however, that unlike earlier models, where matching decisions are based on explicit measurements of feature similarity (e.g., Marr and Poggio, 1979; Jones and Malik, 1992), measurement of similarity in cross-correlation models is conflated with measurement of variability in disparity. Furthermore, similarity biasing only holds if correlation windows are large enough to allow for the presence of local image structures, and if there is no prior image transformation. The local correlation window needs to be large enough to allow sufficient spatial variation in luminance for unambiguous matching, but no so large that it covers regions with widely differing depths (Kanade and Okutomi, 1994). Allowing for image transformations, such as scaling or rotation, prior to cross-correlation could reduce the latter constraint, by allowing for the explicit encoding of local variation in depth. Recently, Vidal-Naquet and Gepshtein (2012) have provided a general approach for the inclusion of image transformations in cross-correlation models of disparity measurement.

Other models, such as those based on the disparity energy model (Ohzawa et al., 1990; De Angelis et al., 1991), apply cross-correlation-like processes, in a manner consistent with the responses of binocular neurons in primary visual cortex. In these models, similarity matching depends upon the use of identical receptive field structures in left and right eyes. The energy model creates disparity selective responses similar to those seen in complex cells in primary visual cortex (V1) by summing the squared responses of pairs of binocular simple cells, arranged in quadrature phase. Disparity tuning is generated in this model through differences in the position, or phase, of left and right eye simple cell receptive fields. Using identical left and right eye receptive field structures for the simple cell components, and summing across spatial location, orientation and frequency channels, allows these energy-based models to be arranged to reflect the cross-correlation of local samples (Fleet et al., 1996; Allenmark and Read, 2011). Physiological evidence suggests, however, that the arrangement of binocular neurons in V1 is subtly different from that required for processing truly analogous to cross-correlation.

As expected in a cross-correlation account of disparity measurement, disparity selective neurons in V1 are fed by left and right eye receptive fields that differ primarily in terms of their relative positions or phases, with very similar tuning to spatial frequency and orientation in the two eyes (Prince et al., 2002a,b). However, differences in orientation tuning for left and right eye receptive fields have been shown in both V1 (Bridge and Cumming, 2001) and V4 (Hinkle and Connor, 2002) binocular neurons. Such cells could encode orientation disparities in a manner that would be useful for the perception of the three-dimensional orientation of surfaces, particularly the slant of surfaces away from the fronto-parallel plane (Greenwald and Knill, 2009) Such neurons would also allow for an encoding of similarity biases more complex than the immediate route offered by existing cross-correlation models.

In addition to potential orientation differences between left and right eye receptive fields, human disparity measurement seems to differ from strict cross-correlation with regards to its use of spatial pooling processes. Psychophysical evidence suggests that larger correlation windows are used for the measurement of larger disparities (Smallman and MacLeod, 1994; Tsirlin et al., 2008; Allenmark and Read, 2011). Additionally, physiological evidence shows that disparity tuning and spatial frequency tuning are correlated (Prince et al., 2002a), with larger disparities detected by neurons with lower spatial frequency tuning. Such coarse-to-fine processing has long been used in computational models of disparity measurement (e.g., Marr and Poggio, 1979; Chen and Qian, 2004) and is implicit in the phase-shift disparity energy model, where disparity tuning is limited by the wavelength of binocular simple cell receptive fields (Fleet et al., 1996; Qian and Zhu, 1997; Chen and Qian, 2004). These findings suggest that the effects of coarse and fine scale manipulations of similarity may deviate markedly from those arising in cross-correlation models of disparity measurement.

Physiological evidence suggesting deviations from disparity measurement through cross-correlation point to a limit in the usefulness of such models as approximations of neural processing. In this paper, we examine this issue by measuring the visual system’s capacity to match ambiguous periodic stereo stimuli using multiple similarity-based matching cues, and compare obtained matching biases to those predicted by a cross-correlation model of disparity measurement. We find that manipulations of luminance and orientation similarity both bias stereoscopic matching, as predicted by the cross-correlation model. However, the effects of orientation differences are smaller than predicted by this model, and the effects of luminance differences larger. Our results are instead consistent with a model of disparity measurement that is biased toward information for binocular matching available at particular spatial frequencies, reflecting the deviations from cross-correlation evident in human visual cortex.

## MATERIALS AND METHODS

### PSYCHOPHYSICAL EXPERIMENT

#### Observers

Psychophysical data was collected for three participants, including both authors. The remaining participant was an experienced psychophysical observer, but was naïve as to the nature of the stimuli, and purpose of the experiment. All participants had normal or corrected-to-normal vision. Participants gave written, informed consent before completing the experiment. Local research ethics boards approved all experimental procedures.

#### Apparatus

Data were collected in Essex and in Stirling. In Essex, the stimulus display and data collection were controlled using a Dell Precision T3600 computer running Windows 7, hosting an NVIDEA Quadro K5000 graphics card, in conjunction with a DATAPixx visual stimulator. Stimuli were presented on a 19 inch Sony Trinitron CRT monitor. Luminance was calibrated using a Minolta LS-100 photometer. The maximum luminance of the monitor was 139.7 cdm^-2^. The spatial resolution of the monitor was 1280 × 1024 pixels and the refresh rate was 100 Hz. The viewing distance was 57 cm. At this distance, 1 pixel subtended 1.6 arcmin.

In Stirling, stimulus display and data collection were controlled using a MacPro computer, with stimuli presented on a 49 cm × 31 cm Apple Cinema HD display. The monitor refresh rate was 60 Hz, with a resolution of 1920 × 1200 pixels. Each pixel subtended 1.1 arcmin at the 76.4 cm viewing distance. The maximum luminance of the monitor was 45.7 cdm^-2^. Luminance was calibrated to vary on a linear scale using a SpyderPro2 calibration device (© Colourworks Inc.).

All stimuli were created using MATLAB in combination with the Psychophysics Toolbox extensions (Brainard, 1997; Pelli, 1997; Kleiner et al., 2007). Dichoptic viewing was achieved using NVIDIA 3D vision liquid-crystal shutter goggles in Essex, and a modified Wheatstone stereoscope in Stirling. Stimulus generation was adapted on each display to maintain identity of angular size.

#### Stimuli

**FIGURE 1:**
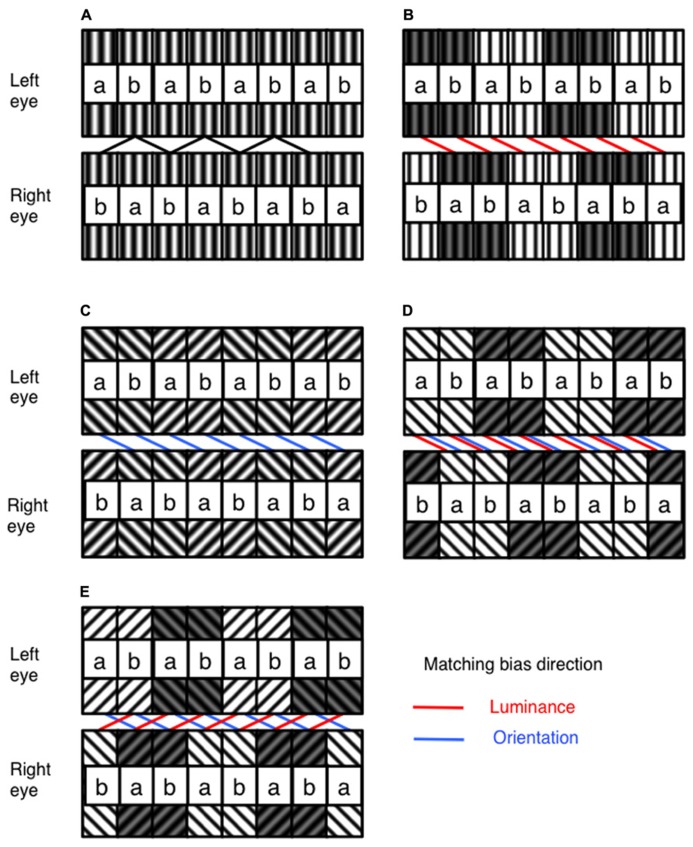
**Illustration of the general structure of the stimulus, and the manipulation of luminance and orientation ambiguity.** **(A)** The stimulus is built from tiles of filtered random dot textures, *a* and *b*. In one eye, these are arranged in a repeating *ab* sequence. In the other eye, they are arranged in a repeating *ba* sequence. With no difference in luminance or orientation, these stimuli can equally be matched with a crossed or uncrossed disparity **(B)** With the addition of a difference in luminance between pairs of *ab* units in each stimulus, matching can be biased toward a particular disparity. **(C)** Similarity in orientation between corresponding *ab* pairings can also be used to bias disparity matching. Luminance and orientation similarity can be introduced so as to bias in **(D)** the same or **(E)** competing directions. The direction of matching based on similarity in luminance and orientation are indicated by the red and blue lines on the figures, respectively. Spatial frequencies for the experimental stimuli were identical across all orientation and luminance similarities, and are shown in this figure for illustrative purposes only. The labeling of *ab* pairings indicated in this figure were not present in actual experimental stimuli.

For convenience, all stimuli will be described in terms of variations in luminance around the mean value, such that the mean luminance is zero and the maximum deviations are ±1. Stimuli were presented such that the range of -1 to 1 was mapped to the full luminance range of the monitor. Ambiguous stereograms were created by concatenating a series of “tiles”, similar to the method used by Goutcher and Hibbard (2010). For each stimulus, two basis tiles (*a* and *b*) were created and were arranged such that different tiles fell on corresponding locations in left and right images (see **Figure 1A**). These tiles were 0.57° wide by 2.27° high. Left and right eye images each contained eight repeats of the *ab* (or *ba*) tile pair, such that the total size of the stimulus was 9.12° wide by 2.27° high. Initially, each pixel was independently assigned a value drawn from a uniform white noise distribution. This was then filtered in the spatial frequency domain, by multiplication with a Gabor (an oriented, two-dimensional sinusoid, windowed by a Gaussian distribution) of standard deviations 6.4 and 19.2 arcmin (orthogonal and parallel to it’s orientation) resulting in noise centered on a spatial frequency of 6.25 cpd. Finally, each tile was windowed by a high exponent Gaussian, in order to remove abutting edges between *a* and *b* tiles. The luminance range of the sample was set to ±0.5 the maximum luminance of the display.

An alternate arrangement of *ab*tile pairs in one eye, and *ba* tile pairs in the other, leads to a stimulus containing ambiguous disparity information. Observers perceive this stimulus as a fronto-parallel surface with either crossed, or uncrossed disparity, equal to the 1.14° size of a single *ab* tile pair. The disparity sign perceived by observers depends upon any prior preference for crossed or uncrossed disparities, combined with any similarity matching bias present in the stimulus. We manipulated image similarity by adjusting the mean luminance of tile pairs, and/or by adjusting the orientation difference prior to filtering. By alternately raising and lowering the mean luminance of tile pairs, similarity matching is biased toward either crossed or uncrossed disparity solutions (see **Figure 1B**).

**FIGURE 2:**
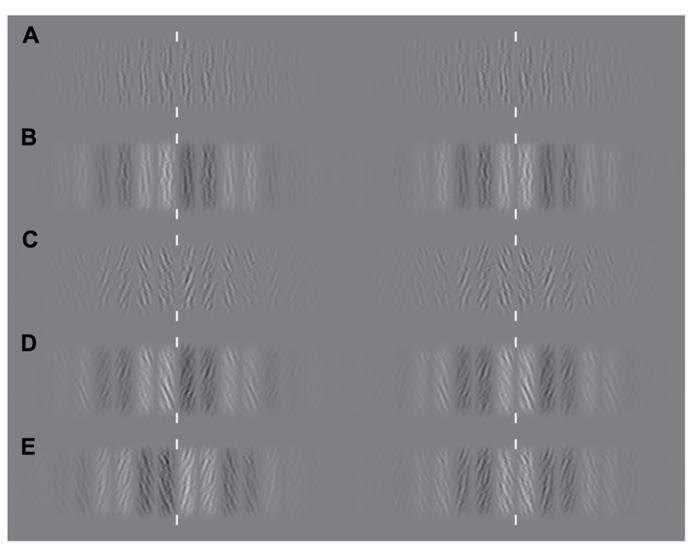
**Examples of the experimental stimuli.** Since stimuli are periodic, either crossed or parallel fusion of left and right columns will demonstrate the effects of manipulating similarity-based matching. White vertical lines indicate the plane of fixation. Examples of experimental stimuli are shown for each case from **Figure 1**. **(A)** An example stimulus containing no luminance or orientation bias. **(B)** Matching is biased through manipulation of luminance similarity only. **(C)** Matching is biased through manipulation of orientation similarity only. **(D)** Manipulations of orientation and luminance similarity are applied in the same direction. **(E)** Manipulations of orientation and luminance similarity are applied in opposite directions.

In **Figure 1B**, raising and lowering the mean luminance of alternate *ab* tile pairs in each eye biases luminance similarity matching toward a crossed disparity solution. Conversely, raising and lowering alternate *ba* tile pairs in each eye will bias luminance similarity matching toward an uncrossed disparity solution. Biases in orientation similarity matching can be produced in much the same way by filtering alternate *ab* or *ba* tile pairs with differently oriented Gabors (see **Figure 1C**). In order to manipulate matching similarity, luminance shifts of 0, ±2, ±4, ±6 and ±8%, and orientation shifts of 0, ±12.25, ±22.5, ±34.75 and ±45° were used. All combinations of luminance and orientation differences were used, resulting in 81 conditions in total, including cases where luminance and orientation similarity were biased in the same or in opposite directions (see **Figures 1D,E**). Examples of the experimental stimuli are shown in **Figure 2**.

#### Procedure

At the beginning of each trial, a central vertical line, of length 16 arcmin, was presented above and below the location of the stimulus. A central fixation cross was also presented. When the observer pressed a response key, the fixation cross was replaced by the stimulus, which was presented for 200 ms before being replaced by the fixation cross. This remained in view until the observer responded, at which point the next stimulus was presented.

The observer’s task was to determine whether the stimulus had crossed or uncrossed disparity, in other words, whether it appeared nearer or further away in depth than the vertical reference lines, which remained on the screen at all times. Responses were made using the computer keyboard. New noise samples were created for every trial. Over the course of five blocks, observers completed 40 trials of each combination of luminance and orientation similarity.

### CROSS-CORRELATION MODEL

**FIGURE 3:**
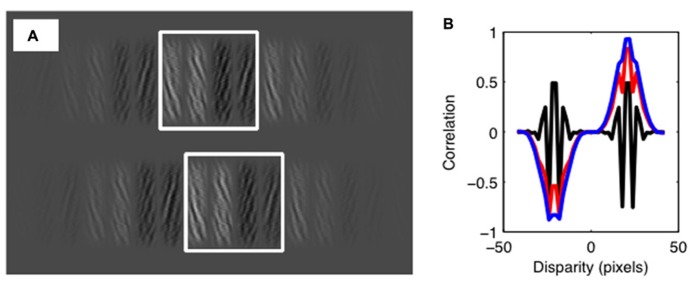
**The cross-correlation model. (A)** Illustration of the correlation window size relative to the experimental stimulus. Using correlation windows greater in size than the range of similarity manipulations ensures that normalization of mean luminance does not degrade model performance. Offset in window positions shows a disparity shift consistent with the periodic structure of the stimulus. **(B)** Output of the standard cross-correlation model. Results show the correlation as a function of disparity. The black line shows the results with no luminance bias. This shows peaks in the correlation function of equal magnitude for crossed and uncrossed disparities. Outputs are also shown for a luminance bias of 0.25 (red line) and 0.5 (blue line). As the luminance bias increases, the correlation increases for one sign of disparity, and decreases for the other. Note that, although the output of the correlation model is shown here at a range of disparities, the decision stage of the model only considers disparities consistent with the periodic structure of the stimulus.

All stimuli used in the psychophysical experiment were analyzed using a cross-correlation model of disparity matching. This local cross-correlation model is widely used as an approximation of the first stages of disparity estimation (Banks et al., 2004; Palmisano et al., 2006; Filippini and Banks, 2009; Allenmark and Read, 2010, 2011; Goutcher and Hibbard, 2010; Vlaskamp et al., 2013). Under this model, a sample patch at a particular location in one image is compared with samples from the other image, as a function of the difference in the sampling location in the two images. Differences in sampling locations are equivalent to disparity, while the level of correlation indicates the similarity between two image samples. When sampling with the correct disparity, the match between the samples, and thus the correlation, will be high. When sampling at an incorrect location, the match will poor, and thus the correlation will be low (see **Figure 3**).

For each stimulus, we compared rectangular samples with crossed and uncrossed disparities the size of one tile of the stereogram. These are the two smallest disparities that provide the best candidate matches for the unbiased stimuli. These were taken from the center of each stimulus image, save for the horizontal shifts required to create the sampling disparities (see **Figure 3**). Below, we report results for a square correlation window with a width of 2.16°. We calculated the cross-correlation between the two samples, *L(x,y)* and *R(x,y)*, given by:

$$C=\frac{\sum_{\left(x,y\right)}\left[L\,\left(x,y\right)-\mu_{L}\right]\,\sum_{\left(x,y\right)}\left[R\,\left(x,y\right)-\mu_{R}\right]}{\sqrt{\sum_{\left(x,y\right)}{\left[L\,\left(x,y\right)-\mu_{L}\right]}^{2}}\,\sqrt{\sum_{\left(x,y\right)}{\left[R\,\left(x,y\right)-\mu_{R}\right]}^{2}}}$$

where, μ_L_ and μ_R_ are the mean luminance of the left and right samples. The psychophysical experiment was simulated, calculating the cross-correlation with a crossed and an uncrossed disparity for each trial, and choosing which of the two had the larger correlation. One hundred trials were simulated for each stimulus configuration presented in the psychophysical experiment. Independent samples of Gaussian white noise, with a mean luminance of zero and a standard deviation of 10%, were added to the left and right eyes’ samples on each trial.

For stimuli such as ours, in which the disparity is constant across the image, using a large correlation window will tend to improve performance. To investigate the effects of spatial scale, rather than using windows of different sizes, we bandpass filtered the images in the Fourier frequency domain. Each stimulus was filtered so as to retain only those components lying within ±1 octave of a central spatial frequency. Central frequencies of 0.47, 0.54, 0.63, 0.76, 0.95, 1.26, 1.38, 1.52, 1.69, 1.90, 2.17, 2.53, 3.04, and 3.79 cpd were used. This simulated the bandpass filtering performed by binocular cells in the primary visual cortex.

## RESULTS

### PSYCHOPHYSICAL EXPERIMENT

**FIGURE 4:**
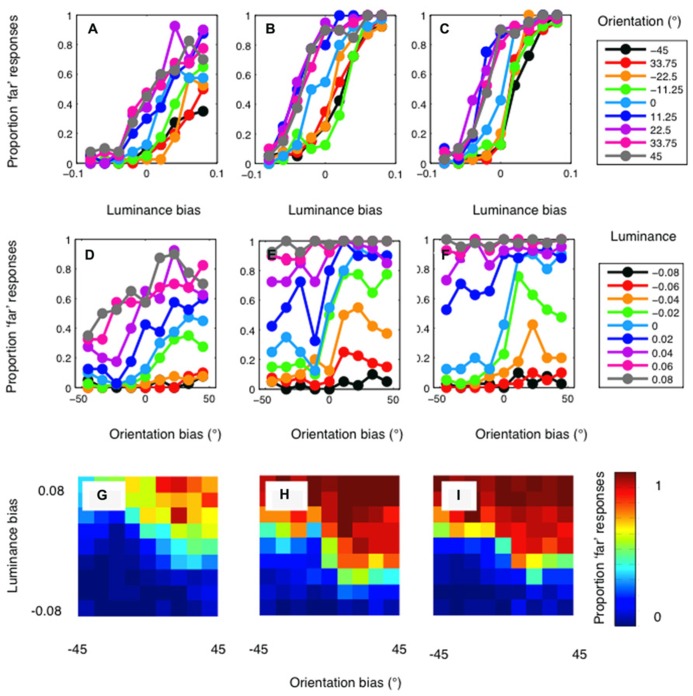
**Psychophysical results for author PH (lefthand column), author RG (central column), and the naïve observer (righthand column).** **(A–C)** show the proportion of “far” results as a function of the luminance bias. Separate lines indicate the results for each level of orientation similarity, as indicated by the legend. A clear effect of luminance similarity is evident for all levels of orientation similarity. **(D–F)** show the results plotted as a function of orientation bias. When matching is not biased by luminance similarity (the cyan curve) a clear effect of orientation is evident for all three observers. With a large luminance matching bias, orientation has little or no effect on disparity matching. **(G–I)** plot the psychophysical data as a heat-map to illustrate the full two-dimensional psychometric function. The color of the pixels indicates the proportion of “far” responses.

The psychophysical results are presented in **Figure 4**. Results are plotted in separate graphs for the three observers. For each observer, a clear luminance bias is evident (**Figures 4A–C**). Orientation also biased matching in the direction predicted (**Figures 4D–F**). However, orientation differences had a rather modest effect on disparity matching. Conversely, manipulations of luminance similarity had a substantially greater biasing effect. In particular, it should be noted that effects of orientation differences were observed only on occasions where luminance biasing was weak (**Figures 4G–I**). Below, we compare these results to simulations conducted with both the standard and modified cross-correlation model.

### CROSS-CORRELATOR MODELS

**FIGURE 5:**
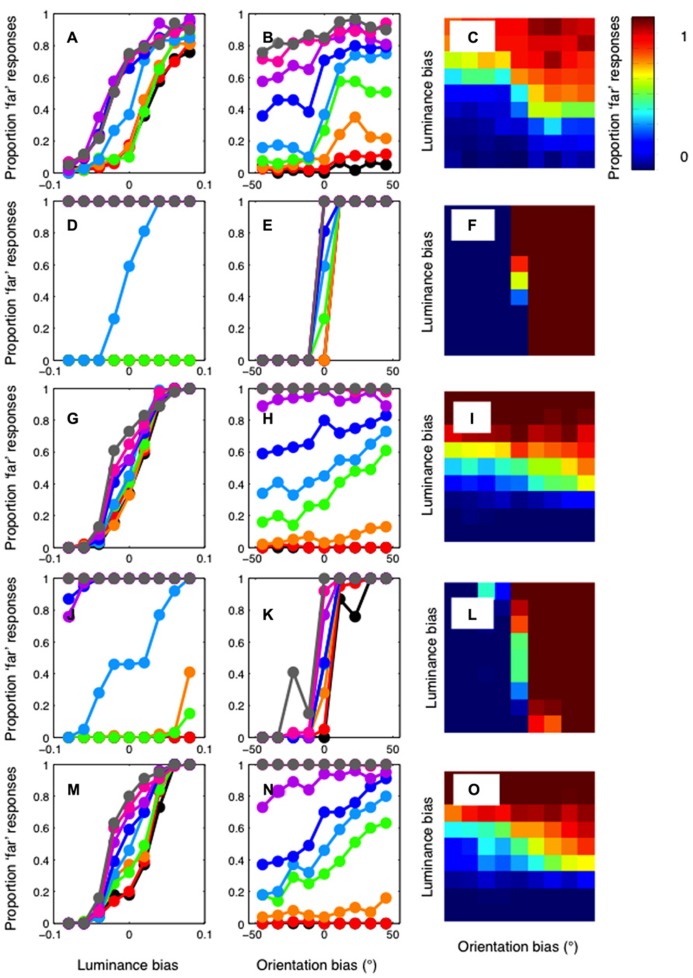
**Results of the cross-correlation model.** Top row: The mean of the psychophysical results plotted in **Figure 4**, across the three observers. Results are plotted **(A)** as a function of luminance bias **(B)** as a function of orientation bias and **(C)** as a two-dimensional heat-map, where color shows the proportion of “far” responses. Second row **(D–F)**: The results of the standard cross-correlation model. Unlike the psychophysical results, the model shows a strong effect of orientation, and an effect of luminance only when there is no orientation bias. Third row **(G–I)**: The results of a cross-correlation model after filtering at a low spatial frequency (0.47 cpd). The model now shows a strong effect of luminance bias, and a weaker effect of orientation bias. Fourth row **(J–L)**: The results of a cross-correlation model after filtering at a high spatial frequency (3.79 cpd). This model shows a strong effect of orientation and a weaker effect of luminance. Fifth row **(M–O)**: The correlation model with filtering at 1.4 cpd shows the closest fit to the psychophysical data.

The results of the standard cross-correlator are shown in **Figure 5**. The mean of the psychophysical results, across the three observers, are plotted in the first row of **Figure 5** as a function of (A) luminance bias, (B) orientation bias, and finally (C) as a function of both. This allows the effect of the luminance and orientation biases to be seen clearly, while also showing the full two-dimensional psychometric function. Equivalent results for the cross-correlation model are shown in **Figures 5D–F**, in the second row. The cross-correlation model shows relatively little effect of luminance similarity matching. Only when orientation similarity provides no biasing signal is any sizeable effect of luminance similarity observable. Conversely, the effects of orientation similarity can be seen at all levels of luminance similarity. Although multiple window sizes were tested for this cross-correlation model, we have reported data only for a window size of 2.16°. Decreasing window size reduces the general effectiveness of both orientation and luminance similarity matching. At the smallest window sizes, no effect of orientation or luminance manipulations is evident.

These results, in which the cross-correlation is calculated using the information available at all spatial scales, are in stark contrast to the pattern of orientation and luminance biasing shown in the human psychophysical data. However, different results are obtained when we calculate the cross-correlation in bandpass-filtered versions of the stimuli. The third row of **Figure 5** shows the results for the model applied after filtering at the lowest spatial frequency (0.47 cpd), in **Figures 5G–I**. Now, a clear luminance effect is evident at all levels of orientation bias. An orientation effect is only evident when the luminance bias is relatively low. The opposite pattern of results is shown when the images are filtered at the highest spatial frequency (3.79 cpd). These results are plotted in **Figures 5J–l**, in the fourth row. Now, a clear effect or orientation is evident at all levels of luminance bias. A clear luminance effect is only evident when there is no orientation bias. We calculated the sum-of-squared-differences between the psychophysical and model results to determine which frequency gave the closest match to our results. The results of the best-fitting model, with a central frequency of 1.4 cpd, are shown in **Figures 5M–O**, in the bottom row. The relatively strong effect of luminance, and a clear, but weaker effect of orientation, is similar to that present in the psychophysical results.

**FIGURE 6:**
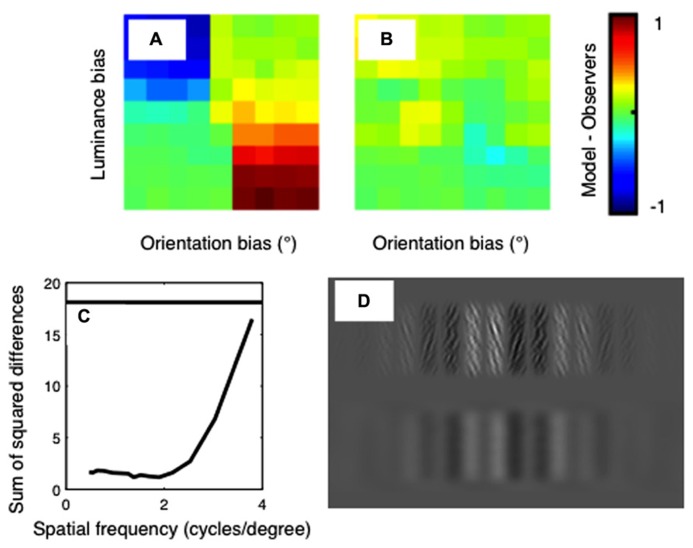
**Differences between the psychophysical data and the model results for (A) the standard correlation model and **(B)** the best-fitting correlation model.** Color indicates the difference in proportion of “far” responses between the models and the averaged human psychophysical data. These differences are summarized in **(C)**, which shows the sum-of-squared differences between the psychophysical and model data as a function of the spatial frequency of filtering. The horizontal line shows the result for the standard cross-correlation model with no filtering. **(D)** An example of a luminance biased stimulus in original (top row) and filtered (bottom row) states. The filter applied has a spatial frequency of 1.4 cpd, which corresponds to that used in the best-fitting model.

The difference between the psychophysical and model results is plotted in **Figure 6**. Results are shown for the standard, unfiltered correlation model in **Figure 6A**, and for the best-fitting model in **Figure 6B**. The sum-of-squared differences between the psychophysical and model results is shown in **Figure 6C**, as a function of the spatial-frequency of the bandpass-filtering applied. The horizontal line on this plot shows the results for the model with no filtering. It is clear from this plot that a good fit to the psychophysical data is obtained when the images are filtered at low spatial frequencies, and the fit becomes poor when the images are filtered at high spatial frequencies.

## DISCUSSION

Our investigation of similarity effects in the perception of ambiguous stereograms has revealed substantial differences between human disparity measurement processing, and the predictions of the standard cross-correlation model. While human observers demonstrate strong effects of luminance similarity manipulations, and relatively weak effects of orientation similarity cues, the standard local cross-correlation model shows an opposing pattern of results. A modified version of this model, which first filters the stimulus in the spatial frequency domain, provides a substantially better match to human psychophysical data. We consider the implications of these results for the encoding of image similarity in disparity measurement. In discussing these issues, we suggest ways in which our findings should constrain models of human stereoscopic matching.

One of the primary advantages of using local cross-correlation as a mechanism for disparity measurement is that it is able to implement many known constraints on stereo matching, without the need for explicit consideration of such rules (Anderson and Nakayama, 1994; Filippini and Banks, 2009; Goutcher and Hibbard, 2010). Cross-correlation models exhibit performance consistent with minimal relative disparity matching (Goutcher and Hibbard, 2010; Vlaskamp et al., 2013), disparity gradient limits (Filippini and Banks, 2009) and coarse-scale luminance and contrast similarity matching (Anderson and Nakayama, 1994; Goutcher and Hibbard, 2010), even though such models do not explicitly apply these rules for matching. Our results show, however, that the similarity-based matching emerging from the cross-correlation model differs markedly from results for human observers. This therefore suggests that the elegant measure of similarity offered by cross-correlation models is not the one used by the human visual system.

Previous research has suggested that deviations from the standard cross-correlation model may be due to initial spatial filtering processes in human vision. For example, Allenmark and Read (2010) showed that human observers perceive surfaces with large sinusoidal variations in depth (surfaces in which depth undulates smoothly) just as readily as they perceive square-waves variations (surfaces in which depth changes abruptly). This is in contrast to the prediction made by their cross-correlation model. Since depth is constant within local regions in squarewave gratings (apart from at their edges), the standard cross-correlation model predicts that observers ought to be better at perceiving square-wave gratings than sinewave gratings, where there are no regions of constant depth. This follows directly from the implicit assumption in cross-correlation models that surfaces are locally fronto-parallel; this assumption is met by squarewave gratings, but not by sinewave gratings (see also, Vidal-Naquet and Gepshtein, 2012). In a later paper, Allenmark and Read (2011) were able to account for their results by proposing a link between the magnitude of disparity, and the size of the correlation window used to match disparity. Such a correlation has been demonstrated in psychophysical results (Smallman and MacLeod, 1994). They argued that large disparities are detected by correlators with large matching windows. If so, then large windows (larger than the regions of constant depth) would be involved in the detection of depth in squarewave corrugations with large disparities. This removes the expected advantage in the perception of square waves, since with larger sampling windows disparity will not be constant across the sample. This example shows the importance of considering the nature of spatial sampling underlying cross-correlation.

The results of our modified cross-correlation model are broadly consistent with this account. While Allenmark and Read (2011) suggest a link between correlation window size and disparity, we consider the related issue of the spatial frequencies to which binocular cells are tuned. If large disparities are preferentially encoded by neurons with large receptive fields, tuned to low spatial frequencies, then similarities and differences at higher frequencies will have limited effects on the disparity matching process. Such a process has clear computational advantages – when matching on a coarse-scale/low frequency (e.g., to detect the location in depth of an object) it is advantageous to ignore depth variations at a finer scale/higher frequency (e.g., those pertaining to the three-dimensional surface structure of the object) that would tend to reduce the matching strength at the correct disparity. Deviations from the standard model may therefore reflect the typical structure of natural scenes, where coarse-scale/low frequency changes are likely to be identical between left and right images, but fine details are subject to greater variation (Li and Atick, 1994).

From a computational standpoint, this account of the pattern of similarity matching in our experiment may arise from two distinct mechanisms. While we have shown that preceding disparity measurement with a spatial frequency filter centered at 1.4 cpd leads to similarity matching biases equivalent to human observers, it is not clear how the visual system makes such selective use of information at this frequency band. One possibility is that disparity measurement depends upon selective use of information at a single spatial frequency channel, or on a weighted combination of multiple channels, where weighting, or channel choice, is contingent on the disparity being signalled. In this case, due to size-disparity correlation, one may expect similarity matching to vary with the disparities available in the stimulus. Alternatively, the coarse-scale/low frequency preferences could arise due to coarse-to-fine matching mechanisms. In this case, one may expect the weight assigned to differing spatial frequencies to remain constant despite changes in the disparity of the stimulus. Whichever mechanism one assumes, however, the selective use of information in particular spatial frequency bands indicates a marked difference from the standard cross-correlation model in human disparity measurement.

While our modified model addresses the role of coarse-scale/low frequency measurements of similarity, a direct role for orientation differences could still be present. The calculation of a cross-correlation from binocular energy neurons assumes that responses are pooled over neurons with identical orientation and frequency tuning in each eye (Allenmark and Read, 2011). However, although binocular neurons are tuned to broadly similar orientations and spatial frequencies in each eye, there is evidence for differences in the exact orientation tuning in the two monocular receptive fields (Bridge and Cumming, 2001). Greenwald and Knill (2009) have argued that the information provided by a system showing such responsiveness to orientation disparities would provide valuable information about the slant of surfaces in depth (see also Vidal-Naquet and Gepshtein, 2012, for a more general approach to handling differences in local binocular image structure). Differences in orientation tuning between the two eyes could therefore reduce the influence of orientation similarity matching, and allow for easier measurement of disparity for such slanted surfaces. The weakness of orientation similarity matching in our experiment could therefore stem from a combination of both the tuning of the visual system to coarse-scale measurements of similarity, and from deviations from the assumption of the standard cross-correlation model that depth is locally uniform.

Given this possible role for direct effects of orientation, an important question remains unanswered. While manipulations of similarity within our stimulus are defined in terms of differences in orientation and in luminance, our modeling results suggest that what is important is not the property that is manipulated, but the scale at which that manipulation occurs. Our manipulations of orientation similarity are comparatively ineffective because they occur at relatively high spatial frequencies. In order to ascertain whether this low frequency bias is a general effect, one would need to examine further similarity manipulations occurring at differing scales. In the specific case of orientation similarity, this is particularly difficult as low frequency orientation differences are likely to result in binocular rivalry, making comparisons of matching similarity difficult. However, previous research has demonstrated that global rivalry impacts on local binocular fusion (Takase et al., 2008), which would seem to be consistent with our account of low frequency dominance in binocular matching.

The suggested departures from the combinations of disparity sensitive neurons required for the implementation of the standard cross-correlation model allow for some simple means for adjusting the relative strengths of different similarity matching dimensions. We have shown that a simple manipulation of spatial frequency can account for the pattern of luminance and orientation similarity matching found in human observers, where the standard model cannot. Similar deviations may also allow for manipulations of the strength of contrast, speed or color similarity matching, should the application of these constraints differ from the predictions of standard cross-correlation.

## Conflict of Interest Statement

The authors declare that the research was conducted in the absence of any commercial or financial relationships that could be construed as a potential conflict of interest.
